# How psychedelic-assisted therapy works for depression: expert views and practical implications from an exploratory Delphi study

**DOI:** 10.3389/fpsyt.2023.1265910

**Published:** 2023-09-28

**Authors:** Lauren Johansen, Paul Liknaitzky, Maja Nedeljkovic, Greg Murray

**Affiliations:** ^1^Centre for Mental Health, Department of Psychological Science, Swinburne University of Technology, Melbourne, VIC, Australia; ^2^Department of Psychiatry, School of Clinical Sciences, Monash University, Melbourne, VIC, Australia; ^3^Turner Institute for Brain and Mental Health, School of Psychological Sciences, Monash University, Melbourne, VIC, Australia

**Keywords:** psychedelic-assisted psychotherapy, mechanisms of action, psychological processes, depression, Delphi study

## Abstract

As investigations into the efficacy of psychedelic-assisted psychotherapy to treat depression continue, there is a need to study the possible mechanisms of action that may contribute to the treatment’s antidepressant effects. Through a two-round Delphi design, the current study investigated experts’ opinions on the psychological mechanisms of action associated with the antidepressant effects of psychedelic-assisted psychotherapy and the ways such mechanisms may be promoted through the preparation, dosing, and integration components of treatment. Fourteen and fifteen experts, including both clinical psychedelic researchers and therapists, participated in Round 1 and Round 2 of the study, respectively. Thematic analysis identified nine important or promising ‘mechanistic themes’ from Round 1 responses: psychological flexibility, self-compassion, mystical experiences, self-transcendence, meaning enhancement, cognitive reframing, awe, memory reconsolidation and ego dissolution. These mechanisms were presented back to experts in Round 2, where they rated ‘psychological flexibility’ and ‘self-compassion’ to be the most important psychological mechanisms in psychedelic-assisted psychotherapy for depression. Strategies or interventions recommended to promote identified mechanisms during the preparation, dosing, and integration components of treatment were nonspecific to the endorsed mechanism. The findings from this study provide direction for future confirmatory mechanistic research as well as provisional ideas for how to support these possible therapeutic mechanisms.

## 1. Introduction

There is increasing evidence to support classic psychedelics with psychotherapy as novel treatments for depression (1). Classic psychedelics are a group of psychoactive substances that includes lysergic acid diethylamide (LSD), psilocybin, *N, N*-dimethyltryptamine (DMT; often consumed as *ayahuasca*) and mescaline. Recent clinical trials have found that psilocybin, ayahuasca and LSD alongside professional support produced significant, rapid reductions in depression (2–7), which in some cases has been sustained at long term follow up (8, 9). Much of this research has delivered *psychedelic-assisted psychotherapy*, whereby psychedelic dosing is paired with several psychotherapy sessions. With a few exceptions, many of the trials to date have had small sample sizes or lacked control groups, although several large-scale randomized controlled trials are currently underway globally. Further, there is growing pressure for these substances to be implemented into clinical practice, with Australia recently approving authorized psychiatrists to prescribe psilocybin to those with treatment resistant depression (10).

As efficacy trials continue and psychedelic-assisted psychotherapy appears closer to being implemented into clinical practice, it is vital that there are simultaneous investigations into *how* psychedelics may be working to alleviate symptoms of depression. Gaining a greater understanding of the underlying mechanisms of action enables treatment outcomes to be optimized through the identification of ‘active ingredients’ of treatment (11). Such components may then be specifically targeted in treatments, while redundant elements are removed (12). This knowledge may extend to the development or modification of other depression therapies, while also contributing to the refinement of theories of depression and increasing the ability to implement preventative measures (11). Mechanistic research can also establish individuals who may be unsuitable for a particular treatment or who are unlikely to respond (13).

Unlike typical pharmacological treatments of depression, psychedelic-assisted psychotherapy generally entails a substantial altered state of consciousness. Participants from clinical trials have reported their acute psychedelic experiences to be personally relevant and meaningful (14), often relating to processes attributed to the maintenance of depression symptoms by the individual (15). These reports may suggest that psychological mechanisms of action may be operating to alleviate depression symptoms within the acute psychedelic experience and beyond. Psychological mechanisms of action are a powerful level of analysis to investigate in antidepressant treatments, due to the possibility of amending and manipulating active treatment components (11, 12). However, there is also notable complexity in investigating such processes in psychedelic-assisted psychotherapy, due to the various intervention types employed in various treatment phases (e.g., preparation, dosing and integration sessions) as well as the challenges in measuring variables associated with altered states of consciousness.

Preliminary findings have provided support for the role of a variety of psychological mechanisms of action for the antidepressant effects of psychedelic-assisted psychotherapy. This research has largely focused on examining the role of the acute psychedelic experience. Findings have suggested that ‘peak’ psychedelic experiences, such as undergoing a *mystical experience* (feeling a sense of unity with others and the universe, transcendence of time and space, intense positive mood, and a sense of ineffability), may mediate the relationship between psychedelic use and reductions depression post therapy (16, 17). Others have investigated more conventional psychological mechanisms, such as *experiential avoidance* (18) and *rumination* (19), with these processes also found to be reduced following psychedelic-assisted psychotherapy. The identification of seemingly dissimilar mechanism types, with some relating to acute experiences and others relating to changes in the psychology of a person, highlights the complexity of conceptualizing how treatments may produce change and has been described in other psychotherapy research [e.g., (20)]. While there is ultimately a need to understand the relationships between various types of mechanism in a change process, studies to date have typically investigated a single mechanism (e.g., mystical experiences) or mechanism type (e.g., variations on the acute psychedelic experience).

There is currently a dearth of investigations into how psychological mechanisms may operate or be addressed across the whole treatment to support lasting antidepressant effects. The current study, therefore, seeks to explore the possible mechanisms of action in psychedelic-assisted psychotherapy for depression across the treatment process through a two-round Delphi technique of experts in the field. The Delphi technique was selected to complement findings of a systematic review on the same topic (under review), as the Delphi technique typically provides a more contemporary depiction of current thinking. This is particularly relevant in fields which are novel and quickly developing, like the psychedelic literature. Furthermore, it enables the exploration of experts’ experiences which may not be included in the available literature, such as those of therapists delivering the treatment. The primary aim of this study was to explore expert views on the psychological mechanisms of action associated with the antidepressant effects of psychedelic-assisted psychotherapy (Aim 1). A secondary aim was to gain expert views on how such mechanisms may be promoted through psychotherapy in the preparation, dosing, and integration components of psychedelic-assisted psychotherapy (Aim 2).

## 2. Methods

To address study aims, a two-step modified Delphi technique utilizing online surveys was adopted. The Delphi technique assumes the opinion of the group is more valid than that of an individual, so seeks to gather the opinions of a group of experts in the field of interest (21). The Delphi method is characterized by four main features:

(1) anonymity, enabling the expression of opinions without influence of the group;
(2) feedback, allowing experts to clarify or alter their opinions from previous rounds;
(3) iteration, permitting the refinement on views based on feedback from prior rounds; and
(4) statistical analysis of responses (22).

Commonly, the method commences with a brainstorming round, whereby experts are able to provide their general opinions on the topic in response to open-ended questions, followed by a round whereby variables are rated on their importance (23). Finally, a third round of ranking variables against one another may be conducted (23). This final step was not conducted here, due to the study’s fundamentally exploratory aims.

### 2.1. Experts

The Delphi method utilizes purposeful, rather than random sampling to recruit identified experts in the chosen field (21). Heterogeneity within experts is considered desirable as it may elicit a wide variety of opinions within the field. In the present study, expert heterogeneity was achieved by compiling lists of international researchers as well as therapists who had been involved in psychedelic-assisted psychotherapy trials. Explicit inclusion criteria were developed to identify individuals with specialized knowledge in the field of psychedelic-assisted psychotherapy for depression: (i) researchers were eligible if they had at least one first or last author publication that was included in a systematic review of the same topic being completed concurrently by the current authors (under review); (ii) therapists were recruited through existing professional networks and considered eligible for participation if they had conducted psychedelic-assisted psychotherapy for depression in the context of a clinical trial.

Following ethical approval from Swinburne University of Technology Human Research Ethics Committee (approval number: 20215758-8858), eligible experts were emailed an invitation to participate in the study. A total of 75 researchers and 15 therapists were invited to participate in the study. The substantial skew toward inviting researchers was due to the ease of identifying relevant researchers compared to therapists. These two groups were collapsed during analysis.

### 2.2. Procedure

**Figure 1:**
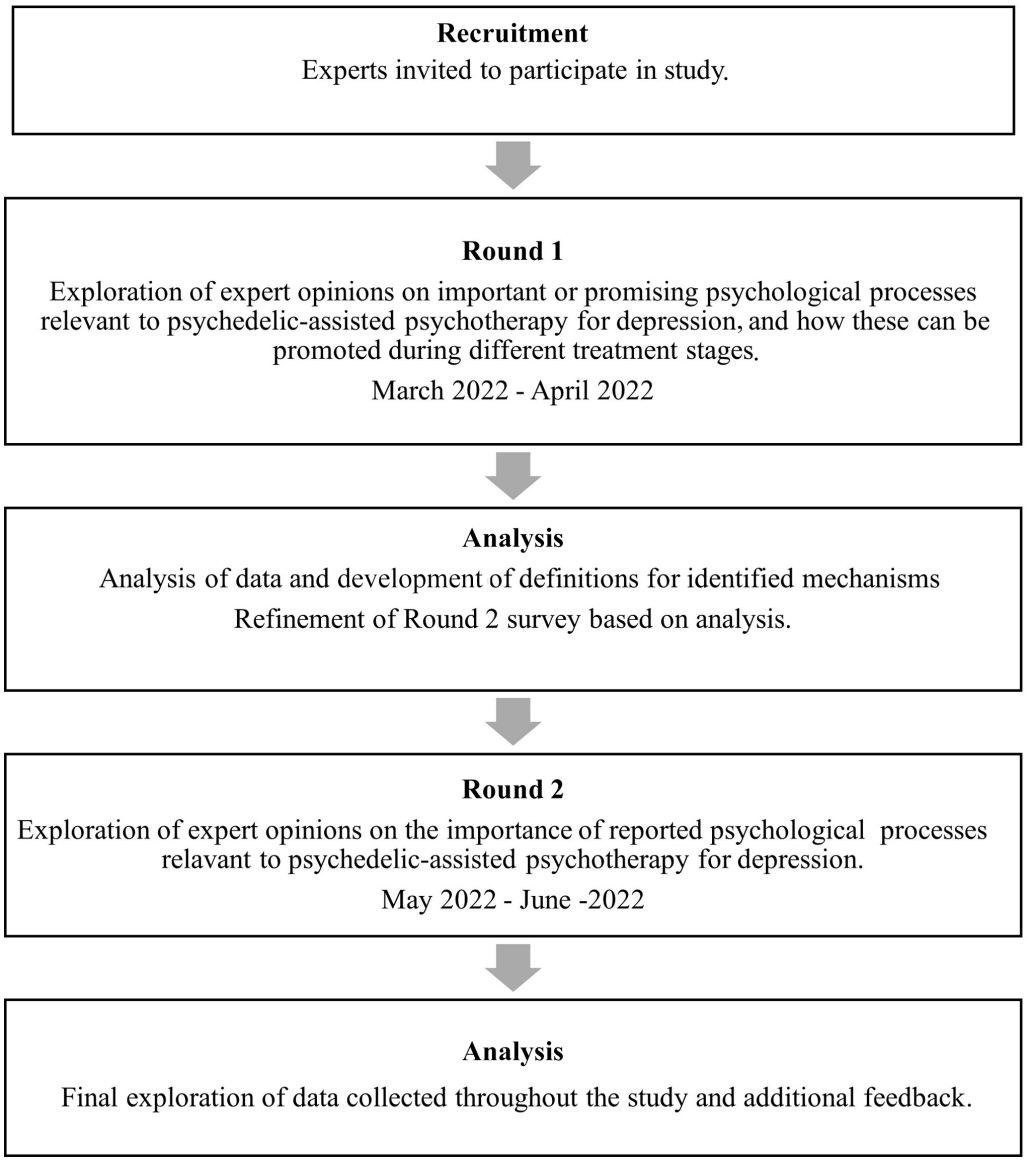
Flow chart of study design.

The study was an iterative process that involved two rounds of surveys administered online through Qualtrics software, version 2022 (Qualtrics, 2022). See Figure 1 for a flow chart of the study design and Supplementary material for the surveys. The aim of Round 1 was twofold: to brainstorm various psychological mechanisms of action that experts believe to be relevant to the antidepressant effects of psychedelic-assisted psychotherapy (Aim 1); and to gain expert views on how such mechanisms may be promoted through psychotherapy across the preparation, dosing and integration components of psychedelic-assisted psychotherapy (Aim 2). Each mechanism was probed individually in a series of items. The online survey permitted participants to loop back to the start to include as many mechanisms as desired. For each mechanism, participants were asked to:

(1) describe a mechanism they believed to be ‘important or promising’ to the antidepressant effects of psychedelic-assisted psychotherapy. In accordance with the study’s exploratory nature, the term ‘promising’ was used to encourage experts to include mechanisms that may not have extant published support (Aim 1);
(2) give their rationale for including the mechanism. To provide guidance, brief examples of possible rationales were offered: “e.g., empirical evidence, theoretical importance, anecdotal or personal experience” (Aim 1);
(3) indicate whether the mechanism was related to a specific classic psychedelic (e.g., ayahuasca, LSD, DMT, psilocybin, mescaline or ‘other’). Experts were able to select as many psychedelics as they desired (Aim 1), and;
(4) describe ways in which this mechanism could be ‘supported or promoted’ during preparation, dosing, and integration sessions (Aim 2).

Experts were then directed to demographic questions relating to their age, gender identity, main country of work, role in psychedelic-assisted psychotherapy, and expertise in working with a specific classic psychedelic.

The Round 2 survey exclusively addressed Aim 1 of the study and intended to rate the mechanisms (derived from the data analysis of Round 1 responses) on importance to the antidepressant effects of psychedelic-assisted psychotherapy. In the Round 2 survey, experts were presented with the mechanisms derived from the analysis of Round 1 responses and were asked to rate the importance of the mechanism on a five-point Likert scale (1 = Not at all important to 5 = Extremely important), alongside an optional reason. A definition was provided alongside the mechanism and experts were requested to rate the importance of the mechanism based on the definition provided to allow for comparison between responses. Demographic questions from Round 1 were then repeated.

### 2.3. Data analysis

The process of analyzing responses relating to Aim 1 of Round 1 was similar to that described by Moynihan and colleagues (24) and Milat and colleagues (25). An inductive thematic analysis was used to interpret the responses to the open-ended questions of mechanisms associated with the antidepressant effects of psychedelic-assisted psychotherapy. These responses were examined, synthesized, and integrated under broad coding themes, with the aim of identifying themes that linked participants descriptions of mechanisms. These themes were then presented back to experts in Round 2 as ‘mechanisms’ and will henceforth be referred to as ‘mechanistic themes.’ An inductive approach was deemed appropriate due to the exploratory nature of the study aims (26). Once finalized, a definition was developed to accompany each mechanistic theme in the Round 2 survey for the sake of clarity. Both *emic* (via participant responses) and *etic* (via accepted definitions from the literature) perspectives were employed in the development of definitions (24). Emic perspectives were utilized when reported themes were not ideas commonly discussed in the literature, or experts had provided clear, specific, and consistent definitions themselves. Etic perspectives were employed when themes had overwhelmingly agreed upon definitions regularly drawn upon in the literature or when experts themselves had directly drawn from accepted theories or relevant psychometric measures.

Qualitative content analysis was used to describe the ways in which each specific mechanism could be promoted during each stage of psychedelic-assisted psychotherapy (Aim 2). This included synthesizing expert responses for clarity and conciseness, as well as removing repetition. Qualitative content analysis seeks to provide literal, descriptive reports of patterns across data, rather than attempting to develop rich meaning or theory from responses (27). This analytic approach was utilized for Aim 2, as the majority of responses were too brief to support more nuanced inferences (28).

Descriptive and frequency statistics were calculated in in IBM SPSS (version 28) to analyze the perceived level of importance of the different mechanisms presented in the survey. Qualitative content analysis was conducted on responses to open ended questions regarding the inclusion of the mechanism as a promising or important mechanism, as well as the corresponding definition. Qualitative content analysis was also conducted on the open-text responses regarding additional mechanisms that were not included in the Round 2 survey that experts considered important to the antidepressant effects of psychedelic-assisted psychotherapy.

## 3. Results

A total of 90 experts (75 researchers, 15 therapists) were invited to participate in the Round 1 survey. Fourteen experts completed the entirety of the Round 1 survey and 15 completed Round 2 (see Table 1 for participant demographics).

**Table 1:** Participant demographics^1^.

	Round 1		Round 2	
	*n*	*%*	*n*	*%*
Gender				
Female	2	14.3	4	26.6
Male	12	85.7	11	73.3
Age				
25–34 years	3	21.4	2	13.3
35–44 years	5	35.7	6	40
45–54 years	2	14.3	3	20
55–64 years	1	7.1	3	20
65+ years	3	21.4	1	6.7
Main country of work*				
USA	6	42.9	5	33.3
UK	2	14.3	4	26.3
Australia	1	7.1	1	6.6
Canada	–	–	1	6.6
France	1	7.1	–	–
Israel	1	7.1	1	6.6
Netherlands	–	–	1	6.6
Portugal	1	7.1	–	–
Switzerland	1	7.1	1	6.6
Germany	1	7.1	-	–
Role in field				
Researcher	7	50	9	60
Therapist	5	35.7	4	26.6
Other**	2	14.3	2	13.4

^1^ Expert’s email addresses were not linked to responses, meaning it was not possible to exclusively contact experts who had fully completed Round 1 when recruiting for Round 2. As such, the Round 2 sample is not exclusively comprised of those who fully completed Round 1. * One participant did not provide a valid response to ‘Main Country of Work’ in Round 2. **Other roles reported included acting as both a researcher and therapist in trials.

### 3.1. Aim 1 results

**Figure 2:**
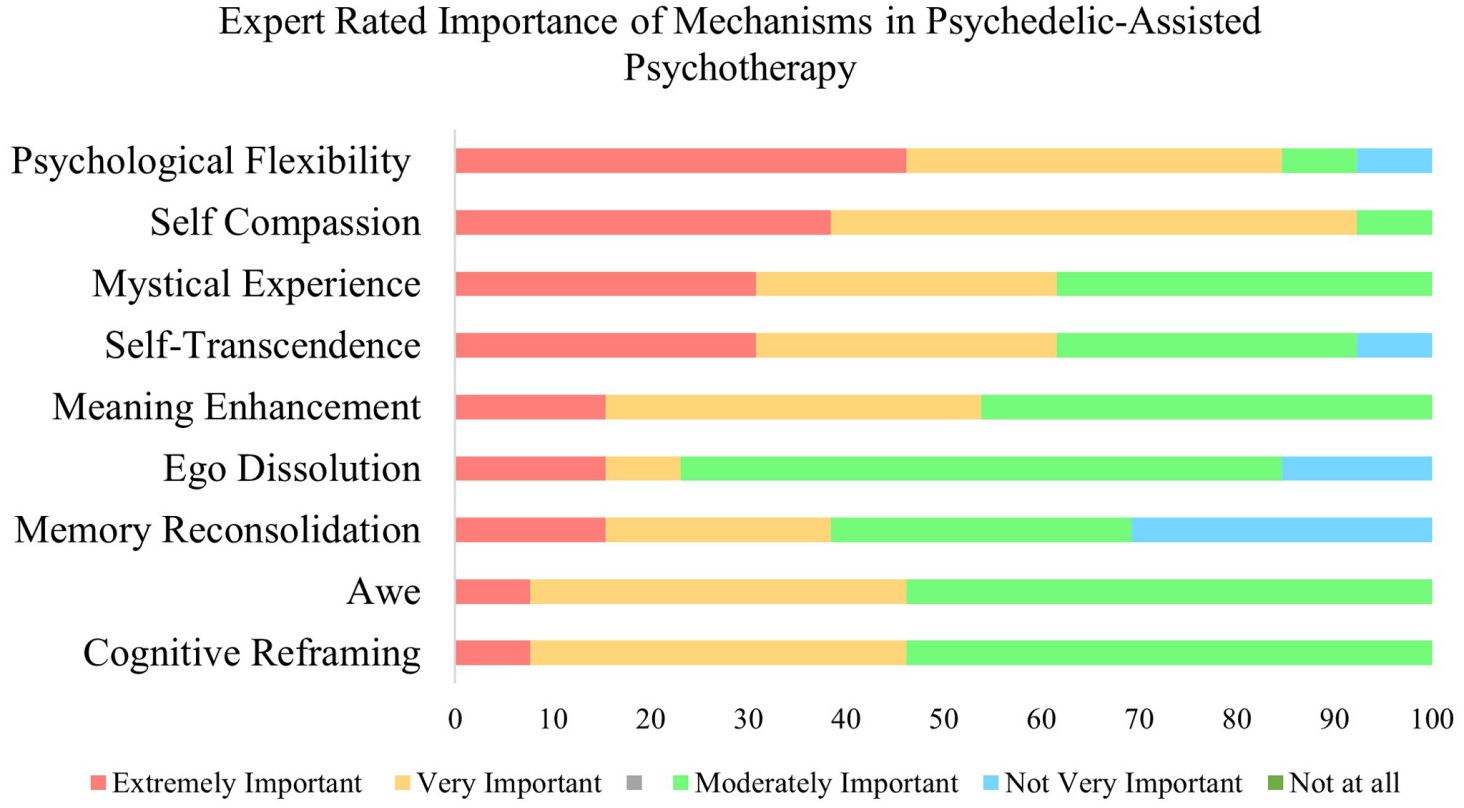
Expert rated importance of mechanisms in psychedelic-assisted psychotherapy.

In Round 1, a total of 27 responses were provided regarding promising mechanisms of action. Eighty percent of responses indicated that the mechanism was not related to a specific psychedelic. Psilocybin and ayahuasca were the only specific psychedelics endorsed to be associated with specific mechanisms. Thematic analysis generated nine themes from responses regarding important or promising mechanisms of action in Round 1. In Round 2, these themes were expressed as mechanisms and presented with definitions back to experts, where they rated each mechanism on its importance to the antidepressant effects of psychedelic-assisted psychotherapy. Psychological flexibility was identified as the most important mechanism, receiving a mean importance rating of 4.32. This was closely followed by self-compassion, which received a mean importance rating of 4.31. See Table 2 and Figure 2 for expert rated importance of mechanisms in the antidepressant effects of psychedelic therapy. Results from Round 1 and Round 2 are presented together below.

**Table 2:** Expert rated importance of mechanisms.

Mechanism	*M*	SD
Psychological flexibility	4.32	0.93
Self-compassion	4.31	0.63
Mystical experience	3.92	0.86
Self-transcendence	3.85	0.98
Meaning enhancement	3.69	0.75
Cognitive reframing	3.54	0.66
Awe	3.52	0.66
Memory reconsolidation	3.23	1.10
Ego dissolution	3.23	0.93

#### 3.1.1. Mechanistic theme one: psychological flexibility

This mechanistic theme encompasses responses which described psychological flexibility and components of the acceptance and commitment therapy (ACT) hexaflex [acceptance, contact with the present moment, cognitive defusion, self-as-context, values and committed action (29)], as responses typically described these facets when elaborating on ‘psychological flexibility’. It was suggested that psychedelics may encourage “being in contact with the present moment, fully aware of emotions, sensations, and thoughts, welcoming them, including the undesired ones, and moving in a pattern of behavior in the service of chosen values.” Another expert suggested that psychedelics may encourage flexibility in “considering both one’s story (major life narrative) [and] expectations of how one should behave in the moment [which may] loosen habitual patterns that maintain depression.” Several experts detailed just one of the six core processes in their response (e.g., psychedelics may “lead to moments of experiencing self-as-context”), which may suggest that some aspects of the ACT model may be considered more relevant than others in the context of psychedelic-assisted psychotherapy.

#### 3.1.2. Mechanistic theme two: self-compassion

The mechanistic theme of self-compassion referred to expert’s descriptions of witnessing participants experience acute self-compassion during the peak psychedelic experience, and for this compassion to continue following treatment. This theme was indicated to be associated with psychological flexibility and memory reconsolidation, with one expert describing psychedelics to lead into “re-experiencing specific, traumatic events, and eventually those experiences softening into moments of self-compassion and alternate perspectives.” Self-compassion was also reported to be important independent of other processes, mechanisms, or themes. During analysis, self-compassion was recognized as a mechanistic theme independent from others due to the reporting of its importance across various response types.

#### 3.1.3. Mechanistic theme three: mystical experiences

Several participant responses were collated under the mechanistic theme of mystical experiences. These responses either directly used the term ‘mystical experience’ or included facets of ineffability (difficulty describing the experience), sacredness, unity with all things and noetic quality (sense of experiencing truth and the ultimate reality).

#### 3.1.4. Mechanistic theme four: self-transcendence

The mechanistic theme of self-transcendence referred to participant descriptions of identifying with something larger than the self. Experts described this experience as potentially reducing the ingrained self-narratives commonly experienced by those with depression. It was suggested that this experience may promote the ‘self-as-context’ principle that is a core component of ACT, as well as being associated with oceanic boundlessness (a term often linked to mystical experiences). Responses under this mechanistic theme differed from those of ego dissolution (see below) in that self-transcendence was discussed as both an experience and a trait which extended beyond the peak psychedelic experience, while ego dissolution was exclusively discussed in the context of the peak psychedelic experience.

#### 3.1.5. Mechanistic theme five: meaning enhancement

Experts described psychedelic engendered meaning enhancement to play an important role in the antidepressant effects of psychedelic therapy. As defined in the literature, meaning enhancement can relate to the amplification of the perceived meaning of objects, activities, relationships, emotions, thoughts and beliefs (30). Specifically relating to the treatment of depression, one participant identified that psychedelics may result in an “amplified significance of … therapeutic insights which emerge during treatment.”

#### 3.1.6. Mechanistic theme six: cognitive reframing

The mechanistic theme of cognitive reframing referred to participant responses describing a change or reframe of an individual’s ingrained cognitions (e.g., thoughts, beliefs, memories, perspectives) about the self, world and others that contribute to the ongoing experience of depression. Responses described the capacity for psychedelic-assisted psychotherapy to encourage “the emergence of new perspectives on existing mental issues and life situations,” which were previously “outside the minds habituated ways of action.” “The discovery of different options [and] patterns of thought and behavior” may apply to “many different areas – worldview, relationships, self-esteem, etc.’. These responses differed from those relating to psychological flexibility as they emphasized altering thoughts, perspective, or beliefs, and not defusion from thoughts.

#### 3.1.7. Mechanistic theme seven: awe

Undergoing an experience of awe was identified as a mechanistic theme in participant responses. Originally presented in the context of psychedelics by Hendricks (31), awe is an emotion suggested to arise in response to coming into contact with *vastness,* which must be *accommodated* through changes to existing knowledge structures (32, 33). Accommodation is required for one to make sense of the encounter with vastness, which may either be perceptual (e.g., witnessing a boundless ocean or large human made structure) or conceptual (e.g., an idea with wide reaching implications). In the current study, participants described awe in a spiritual context, and suggested it could be promoted by ‘exposure to nature, music, art and other stimuli’ while under the influence of a psychedelic.

#### 3.1.8. Mechanistic theme eight: memory reconsolidation

Various participant responses highlighted the relevance of memories, particularly memories of traumatic events, as possibly associated with the antidepressant effects of psychedelic-assisted psychotherapy. Experts indicated that throughout the psychedelic dosing sessions, individuals may re-experience traumatic events and the emotions this event precipitated. This occurrence may lead to further processing of the events and memories being reconsolidated with a more adaptive perspective. One expert suggested that, in this way, psychedelics may act as a catalyst for undergoing exposure-like therapy.

#### 3.1.9. Mechanistic theme nine: ego dissolution

Defined as the disruption to boundaries between the self and world and the increased sense of unity with others and one’s surroundings, ego dissolution is a commonly described mechanism in the literature (34, 35). One participant noted that ego dissolution “is arguably just one sub-measure of mystical-type experiences.” However, as ego dissolution was reported exclusive to mystical experience by some experts and is often discussed in the literature independent of mystical experiences [e.g., (36)], it was decided to distinguish between the two.

### 3.2. Aim 2 results

Experts provided a wide variety of strategies or techniques to promote mechanisms during preparation, dosing and integration. See Table 3. Common responses related to educating participants about peak psychedelic experiences that may occur during dosing, as well as conducting general psychoeducation, rapport building and goal setting. However, due to the small sample size, these strategies should be considered highly exploratory.

**Table 3:** Expert reported strategies for promotion psychological mechanisms of action during preparation, dosing, and integration sessions.

Mechanism	Preparation	Dosing	Integration
Psychological flexibility	Identify clearly defined treatment goals that are collaboratively developed between therapist and participant. Conceptualize treatment objectives using ‘healing’ language, instead of ‘curative’ language. Introduce ACT concepts (e.g., the hexaflex) and strategies. Practice mindfulness and acceptance of challenging emotions	Limit engaging in direct intervention. Be aware of avoidance behaviors. Provide gentle encouragement to promote acceptance and openness to experience. Encourage relaxation techniques to promote present moment awareness. Note areas where psychological flexibility occurs (to discuss in integration)	Explore ‘values’ themes which may have occurred during dosing and integrate these into the participants life. Discuss new perspectives of the self that may have emerged during dosing. Discuss new behaviors that align with functional changes in ‘values. Include defusion and acceptance to promote openness and flexibility. Promote regular mindfulness practice
Self-compassion	Establishing a good therapeutic alliance and build trust. This may enable exploration of difficulties in receiving encouragement or validation. Inner child visualization exercises Somatic strategies to promote calmness when engaging in confronting material Supporting the view that all emotions and parts of the self are welcome	Provide a non-directive compassionate presence Encourage the client to be accepting of the experience Discourage avoidance of difficult emotions Avoid being complicit with the client in the avoidance of difficult emotions or behaviors	Inquire about emotions that may be labeled as undesirable or ‘unpopular’. This may further demonstrate acceptance Encourage journaling of experience Talk therapy to further address self-compassion
Mystical experience	Inform participant that they may undergo a mystical experience Provide education about what the experience could be like Familiarize participant to the room in which dosing will occur Discuss the participants spiritual life, values, and experiences Discuss spiritual materials (e.g., books or music) that the participant feels a connection with and may wish to have with them in the dosing session	Be nondirective Curate a safe and supportive environment Encourage the participant to ‘let go’ and accept whatever experience they are having Discourage fixation on external stimuli Incorporate spiritual materials and music Do not attempt to invoke the experience through the setting you provide -this may result in avoidance of ‘unacceptable’ parts of the self	Discuss and process the experience in a way which welcomes and values the experience Assist participant in developing a therapeutic narrative for their experience Discuss if / how the experience has altered the participants world view Mindfulness practice Engagement with spiritual communities
Self-transcendence	Inform participant that they may undergo self-transcendence during dosing. Provide education about what the experience might be like Provide psychoeducation and exercises which introduce the concept of the ‘observer self’ from ACT, as well as introducing the ‘conceptualized self’ and the challenges that an overattachment to this self can create	Encourage deep noticing, awareness and introspection. Gently guide participant toward an expansive sense of self	Utilize experience of self-transcendence to support the concepts discussed in ACT. Provide further ACT to encourage psychological flexibility
Meaning enhancement	Discuss participant’s symptoms and experiences of depression, including specific changes they wish to experience because of treatment Introduce the concept of being open to changes in perspectives throughout the treatment which may amplify a sense of ‘meaning’	Provide nondirective support which allows personal significance to emerge without therapist interference	Consider and process insights that emerged during dosing
Cognitive reframing	Build rapport and strong sense of trust with participant. Incorporate psychotherapy approaches and strategies that focus on self-narratives, such as CBT or ACT Discuss notable challenges that the client is processes that may be maintaining depressive symptoms, toward increasing accessibility of the issue during dosing. Aim to increase willingness to work with any unpleasant facets of the self that may emerge.	Provide a ‘safe container’ for the participant and support as needed. Demonstrate acceptance toward whatever the participant experiences.	Discuss participant’s perspectives and attitudes prior to dosing and how these may have changed. Consider how alterations in thought, attitudes and beliefs can be maintained and be applied practically (possibly through ACT or CBT strategies) Implement behavioral activation strategies to engage with pleasurable and meaningful activities.
Awe	Introduce personalized materials or experiences that evoke a sense of awe in the participant (e.g., music, art or nature)	Include materials (e.g., music or photos of nature) that are ‘awe inspiring’ to the participant in the dosing room	Encourage further exposure to stimuli (e.g., nature, music, art), which may reinvoke a sense of awe
Memory reconsolidation	Develop strong rapport and trust Education on the possibility of challenging memories being re-experienced during dosing session and therapeutic ways of managing this (e.g., acceptance) Education on stress and the way it may present in dosing session	Encourage acceptance of experience, while also providing a safe environment if the participant feels unable to engage with challenging memories	Encourage journaling about what was experienced during dosing and what it meant for the participant Incorporate somatically informed therapy and strategies (e.g., body scans) to explore how they body may hold trauma
Ego dissolution	Inform participant that they may undergo ego dissolution. Provide education about what the experience could be like	Encourage acceptance and ‘letting go’. Discourage focus on the self	Encourage exposure to awe-inspiring stimuli, including nature, music, art or other personally significant stimuli or experiences.

## 4. Discussion

This study aimed to explore experts’ views on important or promising psychological mechanisms of action that may contribute to the antidepressant effects of psychedelic-assisted psychotherapy, and the ways in which such mechanism may be promoted during the preparation, dosing, and integration components of psychedelic-assisted psychotherapy. To our knowledge, it is the first study to utilize the Delphi technique in the field of psychedelic research. We identified nine mechanistic themes from responses given in Round 1 of the study. As expected, these were varied in type, with some relating to participant experiences during psychedelic dosing, while others described changes in the participant’s perspectives and cognitions. A wide variety of recommendations were provided regarding the ways in which such mechanisms may be promoted during the preparation, dosing and integration components of psychedelic-assisted psychotherapy. Mechanisms were fed back to experts in Round 2, with *psychological flexibility* rated to be the most important mechanism of action for the antidepressant effects of psychedelic-assisted psychotherapy.

### 4.1. Aim 1 findings

The high average rating of importance received for the role of psychological flexibility in the antidepressant effects of psychedelic-assisted psychotherapy is in line with previous research. Psychological flexibility and the components of the ACT hexaflex have been proposed to have relevance in the therapeutic processes of all components of psychedelic-assisted psychotherapy. Even without the inclusion of psychotherapy sessions, one trial in healthy participants found psychological flexibility to correlate with reductions in depression scores following ayahuasca use (37) and similar findings have been reported in a retrospective survey study (38). This has led to suggestions that including a modified version of current ACT protocols may be an appropriate therapeutic modality to pair with psychedelics, as it could assist in engendering further improvements in psychological flexibility and thus, depression (39, 40).

The high importance rating that self-compassion received from experts was an unexpected finding, as self-compassion has received limited attention within the psychedelic literature thus far. However, retrospective surveys of individuals who had previously used a classic psychedelic found increases in self-compassion to be associated with reductions in depression symptoms (41). In addition, interviews with clinical trial participants experiencing cancer-related distress found self-compassion to be a major theme during participant’s psilocybin experience (15), with similar reports from individuals undertaking psilocybin-assisted psychotherapy for alcohol use disorder (42). Several participants from this trial specifically attributed the clinical improvements they had observed to increases in self-compassion (42). These findings and others have led to the recent development of a compassion focused therapeutic protocol for psychedelic therapy (43), which has yet to be implemented in practice. Additionally, there has yet to be mediational analysis published on the relationship between self-compassion and depression change within a psychedelic clinical trial, to the best of our knowledge. Considering findings from the current study, further investigations into the role of self-compassion in the antidepressant effects of psychedelic-assisted psychotherapy are needed.

Several mechanisms relating to peak psychedelic experiences, such as mystical experiences, self-transcendence, and ego dissolution, were reported by experts. Self-transcendence has received considerably less attention than mystical experiences in the current literature, despite there being overlap between the two constructs regarding the experience of unity and connectedness. In the current study, some experts suggested that mystical experiences, self-transcendence, ego-dissolution, and awe all describe a variation of the same peak psychedelic experience. This was despite other experts reporting more than one of these experiences to be important in their Round 1 responses and providing distinct definitions for the various peak psychedelic experiences. Future studies should attempt to characterize the similarities and differences of these overlapping constructs as well as continuing to investigate the role these variables may have on the antidepressant effects of psychedelic-assisted psychotherapy. Experts emphasized the importance of appropriate integration for peak psychedelic experiences, including meaning enhancement and memory reconsolidation, to support antidepressant effects. It was also suggested that such experiences may be counter-therapeutic if resolution did not occur, consistent with previous research (44).

### 4.2. Aim 2 findings

Several important strategies or interventions were identified that may promote the mechanisms identified within the first round of responses. There was substantial overlap between experts in terms of their support for specific methods to promote efficacious psychedelic-assisted psychotherapy, regardless of the primary mechanism they had endorsed. As such, discussion of these strategies here is organized by the phase of treatment they related to, rather than the specific mechanism.

Experts identified several important activities or tasks that should be conducted in the preparation sessions to promote antidepressant effects. Providing education regarding the psychedelic experience and the types of experiences participants may undergo was regularly reported, particularly in the context of the acute psychedelic experience being identified as an important antidepressant mechanism. These responses were in line with the current and historical literature in terms of the emphasis on an individual’s set and their expectations for the psychedelic experience (45). Other responses were more consistent with the typical tasks completed in early sessions of psychotherapeutic interventions, such as history-taking, building rapport, collaborative goal setting and psychoeducation. Some suggested orientating participants to certain psychotherapeutic modalities, such as ACT or cognitive behavior therapy (46), through strategies or techniques commonly employed in such modalities. This included practicing relaxation and mindfulness strategies that participants could draw on during dosing sessions if necessary. Importantly, the inclusion of specific psychotherapeutic techniques within preparation sessions, with the exception of relaxation tools, is not a focal component of the current psychedelic-assisted psychotherapy for depression manuals that have been published [e.g. (39, 40)].

In line with past research, experts overwhelmingly agreed that therapists should predominantly take a non-directive approach during psychedelic dosing sessions, regardless of the mechanism they may be intending to promote. This includes ensuring the participants feel supported and safe throughout the process. Encouraging introspection and discouraging attending to external stimuli was recommended if attempting to elicit a peak psychedelic experience, such as a mystical experience or self-transcendence. There was some disagreement among experts regarding the curation of the setting to elicit such experiences, with some suggesting that incorporating spiritual materials may assist in engendering a mystical experience. However, one expert noted that excessive focus on promoting a ‘spiritual’ experience via a curated setting may inadvertently facilitate participants’ avoidance of addressing personally challenging content. Experts also suggested encouraging acceptance of whatever experience the participant was facing and for therapists to note any avoidance or inflexible behaviors that may occur (for discussion in integration sessions). This was suggested within the context of psychological flexibility, cognitive restructuring, and self-compassion mechanisms. Published manuals on conducting psychedelic-assisted psychotherapy for depression similarly recommend therapists take ‘an attentive but non-intrusive presence’ throughout the dosing session (39).

Integration sessions were reported to be important to both process the acute psychedelic experience and promote long term behavior change. Experts emphasized the importance of developing a therapeutically beneficial narrative of the psychedelic experience, particularly in responses which identified peak psychedelic experiences to be an important mechanism to the antidepressant effects of psychedelic-assisted psychotherapy. Current manuals prescribe an integration session one day following the dosing session to have participants describe their psychedelic experience from beginning to end, with minimal direction from therapists (39, 40). This may assist in committing the experience to memory, as well as commencing the process of the participant connecting insights they may have had during their psychedelic experience to their personal experience of depression. Journaling was also recommended by experts in this study and current manuals as a tool to assist the participant in making both sense and meaning of the experience.

Experts suggested drawing upon the acute psychedelic experience to provide experiential support for processes utilized in conventional psychological modalities (particularly ACT) and thus, long term behavioral change. This may include drawing upon new insights or perspectives from the psychedelic experience into identifying personal values and ways to engage in committed action (47) or discussing the experience of self-transcendence as an (extreme) example of viewing the self-as-context. All other components of the ACT hexaflex (48) were discussed to be important for promoting long term behavior change, as was behavioral activation and cognitive restructuring. Other suggestions included general talk therapy and somatically informed therapy, particularly if the participant has a history of trauma.

### 4.3. Implications for future research

The findings from this study should be investigated by future research for reliability and generalizability. Given the current dearth of research emphasizing self-compassion within the psychedelic literature, and its apparent important as identified by experts in the current study, investigations into the mediational role this plays in depression change following psychedelic-assisted psychotherapy is needed. Similar investigations are needed into psychological flexibility, particularly in the context of a clinical trial with a depression sample. The results of this study also underscore the importance of promoting long-term behavior change following psychedelic therapy, which has not been a major focus of research to date. Incorporating strategies from evidence-based psychotherapies into the psychedelic-assisted psychotherapy model may see further improvements on the efficacy of the treatment model and should be investigated (49). Additional research is also needed into the role of self-transcendence on clinical outcomes. There appears to be overlap between the various forms of the peak psychedelic experience (e.g., mystical experience, self-transcendence, ego dissolution and awe) discussed both within this study and in the literature in general [e.g., (50)], with some experts in this study suggesting that the specific type of the experience may be of less important than developing a therapeutically beneficial narrative of the experience during integration sessions. Further investigation into how these experiences may be similar and different, as well as if a certain type of experience is more influential on clinical outcomes than others, may advance response prediction and the refinement of treatment design. Broadly, there is also a need for investigations into the potential conceptual and/or causal relationships between the mechanisms identified here.

### 4.4. Limitations of the current study

The current study has several limitations that should be taken into consideration when interpreting findings. Firstly, the small sample size and low response rate may mean that the opinions of the experts including in this study are not representative of the opinions of experts in the field of psychedelic research more generally. Secondly, the strategies for promoting certain mechanisms during preparation, dosing and integration were not presented back to experts in Round 2 for discussion, and typically represented a small sample of results. Thirdly, as email addresses were not linked to expert responses, it was not possible to only invite experts to Round 2 who had fully completed the Round 1 survey. Finally, we opted to include all components relating to the ACT hexaflex under the broad term ‘psychological flexibility’ for the sake of brevity. This has impacted the specificity of findings and further investigations into the role of all components of the hexaflex have on clinical outcomes on psychedelic-assisted psychotherapy are needed.

## 5. Conclusion

The current Delphi study has provided insight into the mechanisms experts in the field believe to be important when psychedelic-assisted psychotherapy reduces depression. In addition, this study outlined several approaches that could be employed to support these mechanisms, according to experts. Notably, psychological flexibility was identified by experts to be the most important psychological mechanism for the antidepressant effects of psychedelic-assisted psychotherapy. With respect to professional support, experts highlighted the need to promote long-term behavior-change beyond the dosing session, an insight which has received minimal attention in the current literature. Future studies should conduct confirmatory investigations on the identified mechanisms, as well as investigating the potential clinical utility of therapeutic approaches reported here.

## Data availability statement

The raw data supporting the conclusions of this article will be made available by the authors, without undue reservation.

## Ethics statement

The studies involving humans were approved by Swinburne University of Technology Human Research Ethics Committee. The studies were conducted in accordance with the local legislation and institutional requirements. The participants provided their written informed consent to participate in this study.

## Author contributions

LJ: Conceptualization, Formal analysis, Investigation, Writing – original draft, Writing – review & editing. PL: Conceptualization, Investigation, Writing – review & editing. MN: Conceptualization, Writing – review & editing. GM: Conceptualization, Formal analysis, Investigation, Supervision, Writing – review & editing.
