# Supplementary material for: How psychedelic-assisted therapy works for depression: expert views and practical implications from an exploratory Delphi study
Source: Front Psychiatry. 2023 Sep 28;14:1265910. doi: 10.3389/fpsyt.2023.1265910 (PMC10568016; doi:10.3389/fpsyt.2023.1265910)
Supplement: Supplementary file 1 [file Data_Sheet_1.DOCX]

**Round One Survey**

Q3.1 Please provide your email address so we can contact you to complete the second round of the Delphi study. Your email address will not be used for any other purpose and your survey responses will be kept confidential.

________________________________________________________________

Q4.1
Please describe **one** important or promising **psychological** mechanism of action* relevant to psychedelic-assisted psychotherapy for **depression** that should be prioritised in treatment. Please provide as much detail as possible. You will be provided the opportunity to discuss additional mechanisms later. 
 
* We define a psychological mechanism of action to be a process, event or change which occurs psychologically (e.g. cognitive, emotional/affective, behavioural, perceptual, spiritual) that may cause a reduction in depression symptoms.

________________________________________________________________

________________________________________________________________

________________________________________________________________

________________________________________________________________

________________________________________________________________

Q4.2 What can be done to support or promote this mechanism during the **preparatory sessions** of psychedelic-assisted psychotherapy?

________________________________________________________________

________________________________________________________________

________________________________________________________________

________________________________________________________________

________________________________________________________________

Q4.3 What can be done to support or promote this mechanism during the **dosing session/s** of psychedelic-assisted psychotherapy?

________________________________________________________________

________________________________________________________________

________________________________________________________________

________________________________________________________________

________________________________________________________________

Q4.4 What can be done to support or promote this mechanism during the **integration sessions** of psychedelic-assisted psychotherapy?

________________________________________________________________

________________________________________________________________

________________________________________________________________

________________________________________________________________

________________________________________________________________

Q4.5 Can you please indicate some of your reasons for identifying this mechanism? (e.g. empirical evidence, theoretical importance, anecdotal or personal experience, intuition/hunch, other).
Please include details.

________________________________________________________________

________________________________________________________________

________________________________________________________________

________________________________________________________________

________________________________________________________________

Q4.6 Does this mechanism relate to a specific psychedelic?

- Yes (1)
- No (2)

Q4.7 Which psychedelic?

- Ayahuasca (1)
- Lysergic Acid Diethylamide (LSD) (2)
- N,N-Dimethyltryptamine (DMT) (3)
- Psilocybin (4)
- Mescaline (5)
- Other: (6) __________________________________________________

Q18 Which psychedelic?

- Ayahuasca (1)
- Lysergic Acid Diethylamide (LSD) (2)
- N,N-Dimethyltryptamine (DMT) (3)
- Psilocybin (4)
- Mescaline (5)
- Other: (6) __________________________________________________

Q4.8 Can you think of another promising mechanism of action for the antidepressant effects of psychedelic-assisted psychotherapy that you wish to include?

- Yes (1)
- No (2)

Q5.1 What is your age?

- <24 years (1)
- 25 - 34 years (2)
- 35 - 44 years (3)
- 45 - 54 years (4)
- 55 - 64 years (5)
- 65 years + (6)

Q5.2 Gender: How do you identify?

- Female (1)
- Male (2)
- Non-Binary (3)
- Prefer not to say (4)
- Prefer to self describe (5) __________________________________________________

Q5.3 In which country have you spent the most time working in the field of psychedelic research or treatment?

________________________________________________________________

Q5.4 What has been your predominant role in psychedelic research?

- Researcher (1)
- Therapist/Guide (2)
- Other (3) __________________________________________________

Q5.5 Which psychedelic/s do you have the most knowledge and/or experience of as a treatment for depression? You may select more than one.

- Ayahuasca (1)
- Lysergic Acid Diethylamide (LSD) (2)
- N,N-Dimethyltryptamine (DMT) (3)
- Psilocybin (4)
- Mescaline (5)
- Other (6) __________________________________________________
