# Supplementary material for: How psychedelic-assisted therapy works for depression: expert views and practical implications from an exploratory Delphi study
Source: Front Psychiatry. 2023 Sep 28;14:1265910. doi: 10.3389/fpsyt.2023.1265910 (PMC10568016; doi:10.3389/fpsyt.2023.1265910)
Supplement: Supplementary file 2 [file Data_Sheet_2.DOCX]

Q1.1 How important do you believe a participant undergoing a mystical experience is to the antidepressant effects of psychedelic-assisted psychotherapy?

**Definition:** Mystical experiences are typically thought to include a sense of unity (with all people and things), sacredness (the encounter is holy or sacred), positive mood (joy, blissfulness, ecstasy), transcendence of time and space and ineffability (difficulty to describe). They also have a noetic quality, which is described as a sense of experiencing truth and reality at a fundamental level.

*Stace, W. T. (1960). The teachings of the mystics: Being selections from the great mystics and mystical writings of the world. New American Library.*

- Not at all important (1)
- Not very important (2)
- Moderately important (3)
- Very important (4)
- Extremely important (5)

Q1.2 Do you have any feedback to provide relating to the inclusion of mystical experiences in this list or the definition that has been provided?

Q2.1 How important do you believe a participant experiencing meaning enhancement is to the antidepressant effects of psychedelic-assisted psychotherapy?

**Definition:** The amplification of the perceived meaning of objects, activities, relationships, emotions, thoughts and beliefs. In relation to psychedelic-assisted psychotherapy, this may involve increased meaning conductive with antidepressant effects, such as an increase in the significance of therapeutic insights that emerge during treatment.

*Hartogsohn, I. (2018). The meaning-enhancing properties of psychedelics and their mediator role in psychedelic therapy, spirituality, and creativity. Frontiers in neuroscience, 12, 129.
McMillan, R. M. (2021). Prescribing meaning: hedonistic perspectives on the therapeutic use of psychedelic-assisted meaning enhancement. Journal of Medical Ethics, 47(10), 701-705.*

- Not at all important (1)
- Not very important (2)
- Moderately important (3)
- Very important (4)
- Extremely important (5)

Q2.2 Do you have any feedback to provide relating to the inclusion of meaning enhancement in this list or the definition that has been provided?

­­­­­­­­­­­­ ____________________________________________________________

Q3.1 How important do you believe a participant experiencing self-transcendence is to the antidepressant effects of psychedelic-assisted psychotherapy?

**Definition:** A feeling of connection to something larger than or outside the everyday sense of self. This may result in a loss of ability to distinguish between self and other, whereby the individual is simply aware and accepting of being an integral part of the universe as a whole.

*Cloninger, C. R., Svrakic, D. M., & Przybeck, T. R. (1993). A psychobiological model of temperament and character. Archives of general psychiatry, 50(12), 975-990.
Garcia-Romeu, A., Himelstein, S. P., & Kaminker, J. (2015). Self-transcendent experience: A grounded theory study. Qualitative Research, 15(5), 633-654.*

- Not at all important (1)
- Not very important (2)
- Moderately important (3)
- Very important (4)
- Extremely important (5)

Q3.2 Do you have any feedback to provide relating to the inclusion of self-transcendence in this list or the definition that has been provided?

________________________________________________________________

Q4.1 How important do you believe a participant experiencing ego dissolution is to the antidepressant effects of psychedelic-assisted psychotherapy?

**Definition:** A disruption to boundaries between the self and world, increased sense of unity with others and one’s surroundings.

*Grof, S. (1980). LSD psychotherapy. Pomona, CA: Hunter House.
Nour, M. M., & Carhart-Harris, R. L. (2017). Psychedelics and the science of self-experience. The British Journal of Psychiatry, 210(3), 177-179.*

- Not at all important (1)
- Not very important (2)
- Moderately important (3)
- Very important (4)
- Extremely important (5)

Q4.2 Do you have any feedback to provide relating to the inclusion of ego dissolution in this list or the definition that has been provided?

________________________________________________________________

Q5.1 How important do you believe a participant experiencing***awe*** is to the antidepressant effects of psychedelic-assisted psychotherapy?

**Definition:** An emotion involving intense pleasure and close to fear which includes experiencing *vastness* (a stimulus perceived as significantly larger than the self) and requires *accommodation* (the adjustment of mental structures to integrate new encounter and the sense of enlightenment when new information is assimilated). 

*Hendricks, P. S. (2018). Awe: a putative mechanism underlying the effects of classic psychedelic-assisted psychotherapy. International Review of Psychiatry, 30(4), 331-342.*

- Not at all important (1)
- Not very important (2)
- Moderately important (3)
- Very important (4)
- Extremely important (5)

Q5.2 Do you have any feedback to provide relating to the inclusion of awe in this list or the definition that has been provided?

________________________________________________________________

Q6.1 How important do you believe***psychological flexibility*** is to the antidepressant effects of psychedelic-assisted psychotherapy?

 **Definition:** "Being able to contact the moment as a conscious human being more fully as it is, not as what it says it is, and based on what the situation affords, persisting or changing in behavior in the service of chosen values".

 Psychological flexibility is fostered through six processes: Contact with the present moment: flexible attending to one's experience in the present moment Values: desired qualities for how one wishes to behave on a continuing basis.  Committed action: effective patterns of behaviour which enable one to live according to their values Self-as-context: observing one's thoughts and feelings without over identifying with them Cognitive defusion: mindful awareness of thinking as it occurs, without attachment to thoughts Acceptance: allowing unwanted private experiences, such as thoughts, feelings, memories and urges, instead of avoiding them. 
 *Harris, R. (2019). ACT made simple: An easy-to-read primer on acceptance and commitment therapy. New Harbinger Publications.*
 *Hayes, S. C., Levin, M. E., Plumb-Vilardaga, J., Villatte, J. L., & Pistorello, J. (2013). Acceptance and commitment therapy and contextual behavioral science: Examining the progress of a distinctive model of behavioral and cognitive therapy. Behavior therapy, 44(2), 180-198.*

- Not at all important (1)
- Not very important (2)
- Moderately important (3)
- Very important (4)
- Extremely important (5)

Q6.2 Do you have any feedback to provide relating to the inclusion of ***psychological flexibility*** in this list or the definition that has been provided?

________________________________________________________________

Q7.1 How important do you believe***cognitive reframing*** is to the antidepressant effects of psychedelic-assisted psychotherapy?

**Definition:** Identifying and altering the way situations, experiences, events, ideas, thoughts and emotions are perceived

- Not at all important (1)
- Not very important (2)
- Moderately important (3)
- Very important (4)
- Extremely important (5)

Q7.2 Do you have any feedback to provide relating to the inclusion of ***cognitive reframing*** in this list or the definition that has been provided?

________________________________________________________________

Q8.1 How important do you believe***memory re-consolidation*** is to the antidepressant effects of psychedelic-assisted psychotherapy?

**Definition:** During psychedelic-assisted psychotherapy, emotional, painful or traumatic memories may be recalled and re-consolidated. This process may reduce the fear response associated with the memory. Alternative information may also be integrated into the original memory. 

*Fattore, L., Piva, A., Zanda, M. T., Fumagalli, G., & Chiamulera, C. (2018). Psychedelics and reconsolidation of traumatic and appetitive maladaptive memories: focus on cannabinoids and ketamine. Psychopharmacology, 235(2), 433-445.*

- Not at all important (1)
- Not very important (2)
- Moderately important (3)
- Very important (4)
- Extremely important (5)

Q8.2 Do you have any feedback to provide relating to the inclusion of ***memory re-consolidation***in this list or the definition that has been provided?

________________________________________________________________

Q9.1 How important do you believe increased ***self compassion*** is to the antidepressant effects of psychedelic-assisted psychotherapy?

 **Definition:** The awareness and acceptance of one's negative emotions, cognitions and sufferings in a nonjudgmental manner, with the recognition that these events are part of the shared human experience. 

 *Neff, K. (2003). Self-compassion: An alternative conceptualization of a healthy attitude toward oneself. Self and identity, 2(2), 85-101*.

- Not at all important (1)
- Not very important (2)
- Moderately important (3)
- Very important (4)
- Extremely important (5)

Q9.2 Do you have any feedback to provide relating to the inclusion of ***self compassion***in this list or the definition that has been provided?

________________________________________________________________

Q12.1 Are there additional psychological mechanisms of action that you wish to report?

- Yes (1)
- No (2)

Q12.2 Please describe the psychological mechanism/s of action* relevant to psychedelic-assisted psychotherapy for depression that should be prioritised in treatment that were not included in the previous questions. Please provide as much detail as possible.

* We define a psychological mechanism of action to be a process, event or change which occurs psychologically (e.g. cognitive, emotional/affective, behavioural, perceptual, spiritual) that may cause a reduction in depression symptoms.

________________________________________________________________

________________________________________________________________

________________________________________________________________

________________________________________________________________

________________________________________________________________

Q13.1 What is your age?

- <24 years (1)
- 25 - 34 years (2)
- 35 - 44 years (3)
- 45 - 54 years (4)
- 55 - 64 years (5)
- 65 years + (6)

Q13.2 Gender: How do you identify?

- Male (1)
- Female (2)
- Non-binary (3)
- Prefer not to say (4)
- Prefer to self-describe (5) __________________________________________________

Q13.3 In which country have you spent the most time working in the field of psychedelic research or treatment?

________________________________________________________________

Q13.4 What has been your predominant role in psychedelic research?

- Researcher (1)
- Therapist / Guide (2)
- Other (3) __________________________________________________

Q13.5 Which psychedelic/s do you have the most knowledge and/or experience of as a treatment for depression? You may select more than one.

- Ayahuasca (1)
- Lysergic Acid Diethylamide (LSD) (2)
- N,N-Dimethyltryptamine (DMT) (3)
- Psilocybin (4)
- Mescaline (5)
- Other (6) __________________________________________________
